# Effectiveness of pegfilgrastim prophylaxis in preventing febrile neutropenia during R-FC chemoimmunotherapy for chronic lymphocytic leukemia: A multicenter prospective phase II study

**DOI:** 10.3389/fonc.2023.998014

**Published:** 2023-03-28

**Authors:** Youngwoo Jeon, Duk-Hwan Yang, Suk-Joong Oh, Jin-Hee Park, Jung-Ah Kim, Sung-Young Kim, Chul-Won Choi, Won-Sik Lee, In-Ho Kim, Yeung-Chul Mun, Gi June Min, Ki-Seong Eom, Seok-Goo Cho

**Affiliations:** ^1^ Lymphoma & Cell Therapy-Research Center, Yeouido St. Mary’s Hospital, College of Medicine, The Catholic University of Korea, Seoul, Republic of Korea; ^2^ Institute for Translational Research and Molecular Imaging, The Catholic University of Korea, Seoul, Republic of Korea; ^3^ Department of Hematology-Oncology, Chonnam National University, Hwasun Hospital, Hwasun, Republic of Korea; ^4^ Department of Hematology and Oncology, Hanyang University Seoul Hospital, Seoul, Republic of Korea; ^5^ Department of Hematology and Oncology, Cachon University Gil Medical Center, Incheon, Republic of Korea; ^6^ Department of Hematology and Oncology, St. Vincent’s Hospital, College of Medicine, The Catholic University of Korea, Suwon, Republic of Korea; ^7^ Department of Hematology, Konkuk University Medical Center, Seoul, Republic of Korea; ^8^ Department of Hematology and Oncology, Korea University Guro Hospital, Seoul, Republic of Korea; ^9^ Department of Internal Medicine, Hemato-Oncology, Inje University, Busan Paik Hospital, Busan, Republic of Korea; ^10^ Department of Hematology and Oncology, Seoul National University Hospital, Seoul, Republic of Korea; ^11^ Department of Hematology and Oncology, Ewha Woman’s University, Mok-dong Medical Center, Seoul, Republic of Korea; ^12^ Division of Lymphoma-Myeloma, Catholic Hematology Hospital, Seoul St. Mary’s Hospital, College of Medicine, The Catholic University of Korea, Seoul, Republic of Korea

**Keywords:** prophylaxis, neutropenia, pegfilgrastim, chronic lymphocytic leukemia, R-FC regimen

## Abstract

**Background:**

A chemotherapy of rituximab, fludarabine and cyclophosphamide (R-FC) has been accepted as a promising frontline chemotherapy in selected patients with chronic lymphocytic leukemia (CLL). Although R-FC regimen is a relatively dose-dense regimen and neutropenia incidence is more than 50%, primary prophylactic pegfilgrastim was not fully recommended in the clinical field. Therefore, the study evaluated the prophylactic effectiveness of pegfilgrastim to reduce the incidence of febrile neutropenia associated with R-FC of patients with CLL.

**Patients and methods:**

A single-arm, multicenter, prospective phase II study was designed to assess the efficacy of prophylactic pegfilgrastim. Thirty-four CLL patients were enrolled and analyzed for neutropenia and other related factors, and comparative analysis was performed with historical cohort.

**Results:**

Compared with our historical cohort, incidence of grade 3-4 neutropenia and febrile neutropenia was remarkably reduced during any cycle of chemotherapy (14.7% vs. 48.2% of study cohort vs. historical cohort during C1, 5.9% vs. 65.8% during C2, 12.9% vs. 80.6% during C3, 10% vs. 84.6% during C4, 3.4% vs. 83.6% during C5, and 10.7% vs. 85.7% during C6, p <0.001). Also, cumulative incidence of disrupted chemotherapy was noticeably reduced in study cohort on any cycles of R-FC regimen (8.8% vs. 22.2% of study cohort vs. historical cohort on C2, 9.7% vs. 25.2% on C3, 13.4% vs. 26.9% on C4, 13.8% vs. 45.2% on C5, 17.9% vs. 47.3% on C6, p=0.007). In addition, treatment-related mortality was 5.9%, which significantly reduced compared to 9.6% of our historical cohort (HR 0.64, 95% CI 0.42–0.79, P = 0.032).

**Conclusion:**

Primary prophylactic pegfilgrastim is effective in the prevention of neutropenia/febrile neutropenia, and infection-related mortality during R-FC regimen in patients with CLL.

## Introduction

Chronic lymphocytic leukemia (CLL) is the most common lymphoid malignant disease in adults in western societies, with a prevalence of approximately 4.7 cases per 100 000 population per year, corresponding to more than 20,100 new cases per year (1, 2). The clinical manifestations and course of CLL are highly variable. The prognosis is determined by several independent risk factors, including cytopenia, lymphadenopathy, chromosome abnormalities, and molecular cytogenetic characteristics (2, 3).

In the past 5 years, the development of highly active novel agents have made it possible to target key pathogenetic pathways of CLL, including Bruton’s tyrosine kinase (BTK), spleen tyrosine kinase (SYK), and phosphoinositide 3-kinase (PI3K) inhibitors that disrupt the B cell receptor (BCR) signaling pathway, and venetoclax, an antagonist of the antiapoptotic protein BCL-2 (4, 5). Although targeted agents provide effective disease control with lower toxicity and mutagenicity, CLL has been treated with various chemotherapeutic regimens for more than five decades. The addition of rituximab to the fludarabine and cyclophosphamide (R-FC) chemoimmunotherapeutic regimen transformed CLL therapy (6), providing significant improvement in progression-free survival (PFS) (hazard ratio [HR] 0.56, 95% confidence incidence [CI] 0.46–0.69, *P* < 0.0001) and a favorable overall survival (OS) rate (HR 0.37, 95% CI 0.48–0.92, *P* = 0.01) (2). Therefore, R-FC is still preferred for use in select patients < 65 years old with untreated IGHV-mutated CLL who receive first-line R-FC, which is expected to achieve a PFS of more than of 10 years with the potential for a cure (7).

R-FC chemoimmunotherapy provided excellent long-term survival outcomes in a young and fit group with an overall response rate (ORR) of 90%–95% and rate of complete remission (CR) of 40%–70% as 3-year survival outcomes. However, an increased incidence of hematological complications during R-FC therapy was reported along with these favorable survival outcomes. Grade 3 or 4 neutropenia occurred at a rate of 34%–85%, the incidence of major infections was 2.6%–16% per chemoimmunotherapy cycle, and the minor infection rate was 10%–38% per course (2, 6–8). Based on these data, purine analog-based regimens are considered to be associated with substantially higher rates of myelotoxicity as part of a dose-dense regimen. With this schedule, myelosuppression, in particular severe neutropenia, is an expected major adverse event. In addition, febrile neutropenia is one of the most important clinical symptoms related to infection such as pneumonia or sepsis during chemoimmunotherapy; it is defined as an absolute neutrophil count (ANC) < 1000/mm^3^ and at least one temperature measurement of ≥ 38°C (9, 10). Severe neutropenia and febrile neutropenia are dose-limiting toxicities of myelosuppressive chemotherapy, and may lead to delays in subsequent chemotherapy schedules or a reduction in the relative dose intensity (RDI), which can negatively affect the long-term outcome in patients (11, 12).

The R-FC regimen for CLL was not considered to be associated with a high risk of febrile neutropenia (> 20%) (13). However, the RDI, calculated as the ratio of the dose actually delivered over time to the standard dose intensity (14), must be as high as possible for R-FC regimens to maximize survival outcomes (15). The incidence of hematological adverse events, such as febrile neutropenia, is increased with a high RDI due to strong suppression of bone marrow function (15). There have been few trials of prophylactic granulocyte colony-stimulating factor (G-CFS) administration in patients with CLL while undergoing treatment with the R-FC regimen, although reducing febrile neutropenia during chemotherapy would be as beneficial as successful completion of the R-FC regimen. In addition, although current guidelines advocate G-CFS for prophylaxis against chemotherapy-induced febrile neutropenia in several intensive chemotherapeutic regimens, G-CSF prophylaxis is still underutilized in clinical practice. This prospective trial was performed to evaluate the effects of prophylaxis with long-acting G-CSF given as pegfilgrastim during R-FC treatment for CLL.

## Patients and methods

### Patients

**Figure 1:**
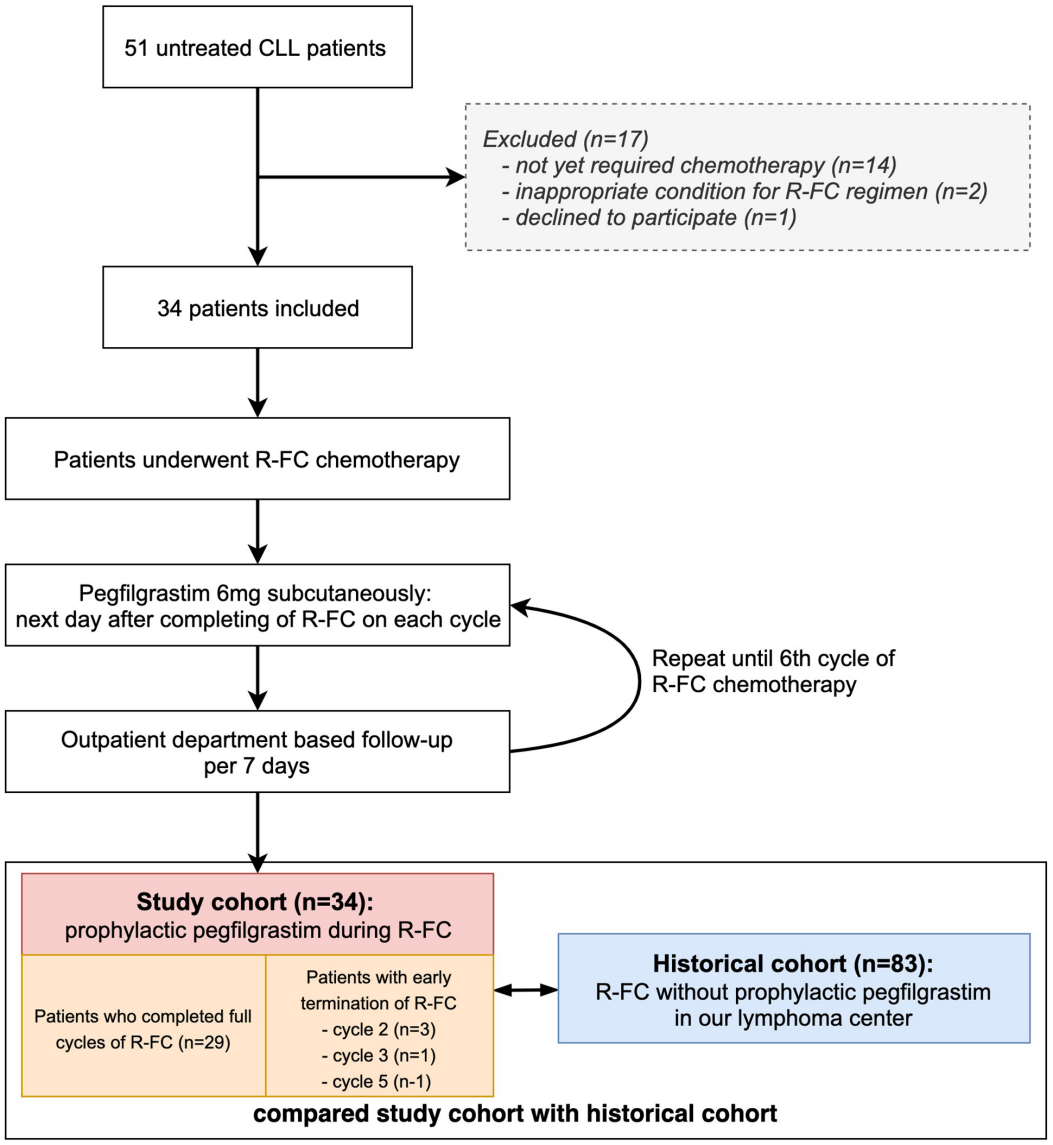
Study flow chart. Flow chart shows patents selection during the study. Study cohort was prospectively enrolled, and historical cohort was retrospectively reviewed in our center.

A total of 51 patients with newly diagnosed untreated CLL between April 2016 and October 2018 were screened for inclusion in this study; 34 patients were enrolled (**Figure 1**). The inclusion criteria were adult patients aged > 18 years; scheduled to receive the R-FC regimen; Eastern Cooperative Oncology Group (ECOG) performance status ≤ 2; and adequate renal function as serum creatine < 1.5 times the upper limit of the normal range; adequate hepatic function as serum bilirubin < 1.5 times the upper limit of the normal range and alanine transaminase (ALT) and alkaline phosphate <3 times the upper limit of the normal range; and cardiac function as no abnormal cardiac wall motion and normal ejection fraction in echocardiography.

Patients were excluded if they had a known grade > 3 hypersensitivity to pegfilgrastim, active uncontrolled infection, HIV-positive patients, active uncontrolled hemorrhagic status, active uncontrolled medical conditions (e.g., decompensated liver failure), severe congestive heart failure, acute myocardial infarction within 6 months before the screening period, Patients with severe or uncontrolled medical conditions (diabetes, chronic heart failure, and severe hypertension), Other clinical trials (chemotherapy, hormone therapy, immunotherapy), women who were pregnant or nursing, and patients who are judged not to be suitable for the progress of the study by the judgment of other clinical trial directors (**Figure 1**).

After chemotherapy cycle 2, although there were no hematological or nonhematological adverse events, three patients discontinued treatment because they did not wish to receive more chemotherapy, and one patient stopped chemotherapy due to a CLL-unrelated cerebral hemorrhage after cycle 3. In addition, one patient abandoned chemotherapy after each of cycles 5 of R-FC due to poor general performance and weakness without hematological toxicity. These five patients were also used in the analysis of the pegfilgrastim effect in this study.

Historical cohort was defined as patients who were diagnosed with CLL by examining the medical records of each institution from January 2013 and treated with R-FC regimen for more than one cycle, and compared with this study cohort.

### Study design and strategy of prophylaxis with pegfilgrastim

This was a multicenter, open-label, non-randomized, non-comparative, phase II study which designed to determine the efficacy and safety with regard to neutropenia/febrile neutropenia of once-per-cycle pegfilgrastim (Neulasta^®^; Kyowa Kirin Korea Co., Ltd., Seoul, Korea) administration during the full course of R-FC regimen in patients with CLL. R-FC chemotherapy was administered as follows: on day 1 of cycle 1, 375 mg/m^2^ of rituximab was administered and 25 mg/m^2^/day of fludarabine with 250 mg/m^2^/day of cyclophosphamide on days 2, 3, and 4. In cycles 2 to 6, the rituximab dose was increased to 500 mg/m^2^ on day 1. Fludarabine and cyclophosphamide were administered on days 1, 2, and 3. Courses were repeated every 4 weeks, depending on the recovery of blood counts, and were delayed until the platelet count exceeded 80 × 10^6^/L and the ANC exceeded 1.0 × 10^9^/L. In addition, the doses of fludarabine and cyclophosphamide were reduced if blood counts had not recovered to the levels described above 5 weeks after the last course of therapy or if major infections occurred. Pegfilgrastim was injected subcutaneously at a dose of 6 mg in a single administration ~24 h after completion of each cycle of R-FC. Blood tests and basic physical examinations were performed every week on an outpatient basis.

All patients underwent a physical examination before pegfilgrastim treatment. If the ANC was ≤ 1.0 × 10^9^/L, blood tests were performed daily until the ANC reached ≥ 1.0 × 10^9^/L on two consecutive days. Body temperature was recorded prior to the first administration and continued daily at the same time until the end of the cycle. Safety assessments were performed within 24 h of chemotherapy. All adverse events were recorded in detail. All patients enrolled in this study underwent antimicrobial prophylaxis with quinolones and azoles, as reported elsewhere (10).

In addition, each endpoint and survival outcomes were compared between this study group and a historical group treated with the R-FC or FC regimen for CLL at our hospital.

The study protocol was approved by the institutional review boards and local ethics committees of all participating centers. All eligible patients gave written informed consent before any study-related procedures. This study was registered with the Clinical Research Information Service (CRIS, https://cris.nih.go.kr, CRIS number: KCT0002087).

### Study endpoints and definition of terms

“Disrupted chemotherapy” was defined as delayed treatment cycles for at least 7 days, and consisted of “time disruption” defined as delayed chemotherapy cycles for at least 7 days if the leukocyte count was < 2,000/mm^3^ before a scheduled cycle, and “dose disruption” defined as reduction of the dose of fludarabine or cyclophosphamide to 25% due to a leukocyte count < 1.0 × 10^9^/L on two consecutive days between cycles (10).

Since the main purpose of this study was to determine whether the use of pegfilgrastim during administration of R-FC chemotherapy could reduce the incidence of grade 3-4 neutropenia: The primary efficacy endpoints were the incidence and duration of grade 3 or 4 neutropenia, defined as an ANC < 1.0 × 10^9^/L. The secondary efficacy endpoints included the incidence of febrile neutropenia defined by an ANC < 1.0 × 10^9^/L and at least one body temperature measurement ≥ 38°C, time to ANC recovery, incidence of disrupted chemotherapy, and the incidence of infection, such as pneumonia, sepsis, or cystitis. The safety and adverse events were assessed using preferred terms designated by the NCI Common Terminology Criteria for Adverse Events (version 5.0).

### Sample size estimation

The sample size of this clinical study was based on hypothesis testing, type I error, and power considerations. The incidence rate of febrile neutropenia in the preemptive treatment group was entered as 1% and the incidence rate in the standard treatment group as 10%, 15%, and 20%, respectively, and was calculated. As a result, at least 31 patients were calculated, and a plan was made to recruit a total of 35 or more to consider a dropout rate of 10%. Incidence of neutropenic fever was calculated using the efficiency–one proportion vs. a given proportion: conditional method.

### Statistical analysis

For endpoint assessment, the difference in grade 3 or 4 neutropenia duration was estimated using the paired *t* test. The least squares mean difference and 95% confidence interval (CI) between two groups were determined. Secondary endpoints were derived from two-sided stratified and non-stratified log-rank tests for time-to-event outcomes. In these analyses, *P* < 0.05 was taken to indicate statistical significance. For safety analyses, the number and ratio of patients experiencing adverse events were summarized, and comparisons between groups were performed using the Fisher’s exact test and χ^2^ test for categorical variables and the Mann-Whitney test for continuous variables. OS was calculated from the date of the start of R-FC cycle 1 until the date of death from any cause or the last follow-up. disease-free survival (DFS) was defined as the time from the date of the start of R-FC cycle 1 until the date of disease relapse/progression, death from any cause, or the last follow-up. The OS and DFS rates were calculated using the Kaplan-Meier survival method with log-rank analysis. The cumulative incidence of relapse (CIR) was calculated with relapse or death from other causes defined as adverse events using the Gray test for univariable analysis and the Fine-Gray method for proportional hazard regressions. All statistical analyses were performed using R version 3.2.0 (Comprehensive R Archive Network, http://cran.us.r-project.org) with the EZR graphical user interface by Y. Kanda (Saitama Medical Center, Jichi Medical University, Saitama, Japan) (16).

## Results

### Patient characteristics

The clinical baseline characteristics of all patients in the study cohort are summarized in **Table 1**. A total of 34 patients with first-line R-FC were included in this study. In 186 cycles of R-FC chemotherapy performed in the study cohort, the median age at the start of cycle 1 of R-FC was 60 years old and slightly more than half of the patients were male. Approximately half of the patients (*n* = 16, 47.1%) were in an advanced Rai stage (stage III or IV) and the majority of patients (*n* = 30, 88.2%) were in a moderate- to high-risk Binet stage (stages B and C). Although the number was small (*n* = 3, 8.8%), the cohort included patients with poor prognostic factors, such as del(17p), which are known to have negative impacts on survival outcomes.

**Table 1:** Patient’s baseline characteristics.

Factor	Study cohort (n=34)	Historic cohort (n=83) *	*p-value*
Age, median (range)	60 (28-81)	59 (24-80)	0.866
Gender, male (%)	20 (63)	58 (70)	0.034
Rai stage, n (%)			0.106
0	2 (5.8)	4 (4.8)	
I	7 (20.6)	15 (18.1)	
II	9 (26.5)	19 (22.9)	
III	9 (26.5)	24 (28.9)	
IV	7 (20.6)	21 (25.3)	
Binet stage, n (%)			0.329
A	4 (11.8)	7 (8.4)	
B	22 (64.7)	59 (71.1)	
C	8 (23.5)	17 (20.5)	
ECOG, n (%)			0.098
0	5 (14.7)	22 (26.5)	
1	29 (85.3)	49 (59.0)	
2	0	12 (14.5)	
Lab data at initial diagnosis			
WBC count, x 10^9^/L, median (range)	52.58 (1.12-528.0)	42.92 (0.98-489.6)	0.109
Hb, g/dL, median (range)	11.3 (7.4-14.5)	10.6 (6.9-15.9)	0.411
Platelet, x 10^9^/L, median (range)	146 (10-435)	136 (9-595)	0.265
LDH > UNL, n (%)	14 (41.2)	33 (39.8)	0.102
β2-microglobulin, median (range)	2.87 (1.08-5.24)	3.12 (0.98-6.35)	0.217
*IGHV*, n (%)^†^			
mutated	34 (100)	34/45 (75.6)	0.098
unmutated	0	11/45 (24.4)	
Del(17p) by karyotyping, n (%)	3 (8.8)	4 (4.8)	0.638
Lines of R-FC chemotherapy, n (%)			0.060
frontline chemotherapy	34 (100)	71 (85.5)	
salvage chemotherapy (2nd/3rd line)	0	12 [10/2 (14.5)]	
Purpose of G-CFS, n (%)			<0.001
prophylactic (pegfilgrastim)	34 (100)	0	
therapeutic (filgrastim)	0	83 (100)	

ECOG, Eastern Cooperative Oncology Group; WBC, white blood cell; Hb, hemoglobin; LDH, lactate dehydrogenase; ULN, upper normal limit; IGHV, immunoglobulin heavy-chain variable; G-CSF, granulocyte colony-stimulating factor; R-FC, rituximab plus fludarabine and cyclophosphamide.

*historic cohort meant patients who had been treated in our center.

†calculated only by patients with recorded data.

For an indirect comparison, the study cohort enrolled in this research was compared with the historical cohort that had been treated in the past by these institutions. There was no difference in the median age between the two groups as about 60 years old (p=0.866), but the male proportion in the study cohort was 63%, which was smaller than 70% of the historical cohort (p=0.034). In addition, there was no difference between the two groups in laboratory test including of white blood cells, hemoglobin, platelets and LDH etc. as well as Rai/Binet stage at initial diagnosis. All patients in the study cohort were previously administrated prophylactic pegylated filgrastim, and patients with historical cohort received non-pegylated filgrastim for the therapeutic purpose of neutropenia (**Table 1**).

### Grade 3 or 4 neutropenia and febrile neutropenia

The incidences of grade 3 or 4 neutropenia in each cycle of chemotherapy are listed in **Table 2**. During the first cycle of the R-FC regimen, five patients had grade 3 or 4 neutropenia with a median recovery time of 6 days. Of these, two patients experienced progressive neutropenia with a fever. In addition, neutropenia occurred in two patients in cycle 2, four patients in cycle 3, three patients in cycle 4, one patient in cycle 5, and three patients in cycle 6. Consequently, among the patients administered pegfilgrastim prophylaxis in a total of 186 cycles of chemotherapy, 9.7% (*n* = 18) showed a median of 4 days of neutropenia. Febrile neutropenia was most common in cycle 1 of chemotherapy, and four cases (2.2%) showed a median of 4 days of febrile neutropenia out of the total of 186 cycles of chemotherapy (**Table 2**).

**Table 2:** Grade 3-4 neutropenia and Febrile neutropenia.

Factor	C1 (n=34)	C2 (n=34)	C3 (n=31)	C4 (n=30)	C5 (n=29)	C6 (n=28)	Sum (n=186)
Grade 3-4 neutropenia							
number of patients, n (%)	5 (14.7)	2 (5.9)	4 (12.9)	3 (10.0)	1 (3.4)	3 (10.7)	18 (9.7)
duration, day, median	6	1	4	2	4	4	4
Febrile neutropenia, n (%)							
number of patients, n (%)	2 (5.9)	0	1 (3.2)	0	1 (3.4)	0	4 (2.2)
duration, day, median	7	0	2	0	2	0	4

C; cycle of chemotherapy, CI; cumulative incidence.

### Cumulative incidence of disrupted chemotherapy

Overall, in the cumulative incidence of disrupted chemotherapy at the final cycle (cycle 6), five patients (17.9%) had a disrupted chemotherapy schedule due to severe neutropenia or febrile neutropenia (delayed recovery of neutropenia, delayed negative conversion of systemic bacteremia, and delayed recovery from various infections). Disrupted chemotherapy consisted of time-disrupted and dose-disrupted chemotherapy; the details of each cycle are presented in **Table 3**. All of these patients were hospitalized for each infection and received intravenous antibiotics with full resolution of infectious episodes. There were no patients in whom chemotherapy had to be stopped due to pegfilgrastim-related adverse events.

**Table 3:** Clinical causes of ‘Disrupted chemotherapy’.

Factor	C1 (n=34)	C2 (n=34)	C3 (n=31)	C4 (n=30)	C5 (n=29)	C6 (n=28)
Cumulative incidence of disrupted chemotherapy, n (%)	-	3 (8.8)	3 (9.7)	4 (13.3)	4 (13.8)	5 (17.9)
Time-disrupted (chemotherapy schedule’s delay)	–	2 (5.9)	2 (6.5)	2 (6.7)	2 (6.9)	3 (10.7)
Delayed recovery of neutropenia		2	2	2	2	2
Delayed negative conversion of bacteremia		0	0	0	0	0
Delayed recovery of infection *		0	0	0	0	1
Dose-disrupted (dose reduction)	–	1 (2.9)	1 (3.2)	2 (6.7)	2 (6.9)	2 (7.2)
Delayed recovery of neutropenia		0	0	0	0	0
Delayed negative conversion of bacteremia		1	1	1	1	1
Delayed recovery of infection *		0	0	1	1	1

The types of infections associated with this study: pneumonia, acute pyelonephritis, urinary tract infection, fever of unknown origin.

### Comparison of this study cohort and our historical cohort

Although this was a single-arm study to determine the efficacy of pegfilgrastim for CLL with the R-FC regimen, indirect comparisons were performed with historical data from CLL patients who had been treated previously at our hospital to determine differences in efficacy with a nonprophylaxis group. As the use of prophylactic pegfilgrastim after R-FC was not approved according to national reimbursement standards, it could not be used previously; therefore, in cases in which the ANC decreased below 1.0 × 10^9^/L, filgrastim was administered for therapeutic purposes after R-FC infusion in the historical cohort.

**Figure 2:**
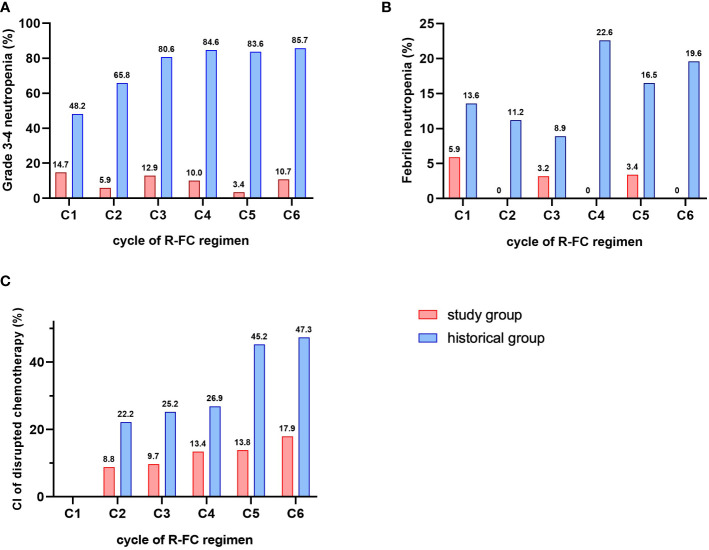
Comparison of G-CSF related-factors. **(A)** incidence of grade 3-4 neutropenia. **(B)** incidence of febrile neutropenia. **(C)** cumulative incidence of disrupted chemotherapy.

The incidence of grade 3 or 4 neutropenia was markedly reduced in the study cohort compared to the historical cohort without pegfilgrastim prophylaxis during any cycle of chemotherapy (14.7% vs. 48.2%, respectively, in cycle 1; 5.9% vs. 65.8%, respectively, in cycle 2; 12.9% vs. 80.6%, respectively, in cycle 3; 10% vs. 84.6%, respectively, in cycle 4; 3.4% vs. 83.6%, respectively, in cycle 5; and 10.7% vs. 85.7%, respectively, in cycle 6, *P* < 0.001) (**Figure 2A**). The incidences of febrile neutropenia were significantly lower in the study cohort than the historical cohort during all cycles of chemotherapy (5.9% vs. 13.6%, respectively, in cycle 1; 0% vs. 11.2%, respectively, in cycle 2; 3.2% vs. 8.9%, respectively, in cycle 3; 0% vs. 22.6%, respectively, in cycle 4; 3.4% vs. 16.5%, respectively, in cycle 5; and 0% vs. 19.6%, respectively, in cycle 6, *P* = 0.004) (**Figure 2B**). Moreover, the cumulative incidence of disrupted chemotherapy was markedly reduced in the study cohort compared with the historical cohort during all cycles of chemotherapy (8.8% vs. 22.2%, respectively, in cycle 2; 9.7% vs. 25.2%, respectively, in cycle 3; 13.4% vs. 26.9%, respectively, in cycle 4; 13.8% vs. 45.2%, respectively, in cycle 5; and 17.9% vs. 47.3%, respectively, in cycle 6, *P* = 0.007) (**Figure 2C**). Hematologic adverse event other than neutropenia were investigated, and thrombocytopenia, anemia, autoimmune hemolytic anemia, and tumor lysis syndrome were not different between the two groups, but major infection rate was statistically significantly less occurred in the study cohort (1.1% vs. 6.1% in study cohort vs. historical cohort, *P* = 0.041, **Table 4**).

**Table 4:** Incidence of grade 3 and 4 adverse events.

	Study cohort (n=34)	Historical cohort (n=84)	p-value
Total no. of patients with at least one grade 3 or 4 event	35.3%	79%	0.029
Neutropenia	9.7%	74.7%	<0.001
Thrombocytopenia	20.6%	19.0%	0.787
Anemia	8.8%	7.1%	0.312
Autoimmune hemolytic anemia	2.9%	1.2%	0.890
Tumor lysis syndrome	0	1.1%	0.233
Infection, major*	1.1%	6.1%	0.041

*Pneumonia, acute pyelonephritis, urinary tract infection, fever of unknown origin.

### Overall survival outcomes and early mortality rate

This was a phase II prospective study conducted without a placebo control group from the beginning. However, it was necessary to compare survival outcomes using the historical cohort from our center to determine the efficacy of pegfilgrastim prophylaxis for neutropenia in a cohort of the same ethnicity treated with a similar therapeutic strategy. The median follow-up period from the start of cycle 1 of R-FC was 2.6 years (range: 3 months to 3.7 years) in the study cohort and 5.9 years (range: 2 months to 14.2 years) in the historical cohort. There were generally fewer deaths and adverse events among patients receiving R-FC. When examining the rate of early mortality, defined as all-cause mortality during the chemotherapy period, it was 5.9% (2/34) in the study cohort, which was significantly reduced compared to 9.6% (8/83) of the historical cohort (HR 0.64, 95% CI 0.42–0.79, *P* = 0.032).

**Figure 3:**
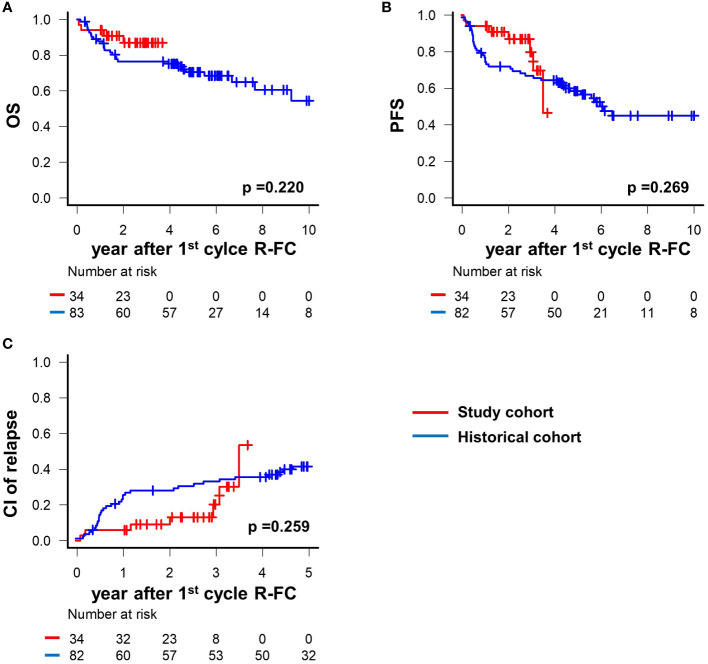
Survival outcomes according to prophylaxis pegfilgrastim. **(A)** overall survival between prophylactic pegfilgrastim and nonprophylaxis group. **(B)** progression-free survival between prophylactic pegfilgrastim and nonprophylaxis group. **(C)** cumulative incidence of relapse between prophylactic pegfilgrastim and nonprophylaxis group.

In addition, to confirm that this reduction in early mortality is related to the mid- to long-term survival outcomes, we conducted an analysis of survival rate for about 3 years. The OS and PFS of the patients at 3 years after the start of cycle 1 of R-FC were 86.4% and 73.6%, respectively in this study cohort (**Figures 3A, B**). There were no significant differences in rates of OS, PFS, or CIR between the present study cohort and the historical cohort (hazard ratio [HR] 1.941, 95% confidence interval [CI] 0.660–5.706, *P* = 0.220; HR 1.593, 95% CI 0.692–3.667, *P* = 0.269; HR 3.240, 95% CI d0.532–3.945, *P* = 0.259, respectively) (**Figure 3**).

## Discussion

In this prospective study, we investigated the role of primary prophylaxis with pegfilgrastim as long-acting G-CSF in a cohort of 34 patients with CLL receiving R-FC. The incidence of grade 3 or 4 neutropenia was 9.7% (18/186 cycles) and that of febrile neutropenia was 2.2% (4/186 cycles). In addition, the cumulative incidence of disrupted chemotherapy was 8.8% at cycle 2 and reached 17.9% at the last cycle (cycle 6) of chemotherapy. These results showed that the incidences of grade 3 or 4 neutropenia and febrile neutropenia were significantly decreased in comparison with our historical cohort treated without pegfilgrastim prophylaxis. In addition, pegfilgrastim prophylaxis reduced the rate of a delayed chemotherapy schedule and dose reduction due to neutropenia. Although it has already been reported that the use of pegfilgrastim for primary prophylaxis rather than filgrastim reduced rates of febrile neutropenia, febrile neutropenia-related hospitalization, and intravenous antibiotic use in patients with breast cancer (17), there have been no studies on the prophylactic use of pegfilgrastim in R-FC regimen for CLL patients. In FLAIR trial (18), non-peglyated G-CSF (as per standard dosing) for days 7 to 13 was recommended for all subsequent cycles of rituximab for participants who have had to have a previous dose delay due to neutropenia. However, any events of infection were occurred in 33.6%, 19.8% of grade 4 hematologic adverse events. Considering previously reported data and this study, it can be assumed that it is more efficient to prevent neutropenia by using pegylated G-CSF prophylactic than non-pegylated G-CSF used after neutropenia has occurred.

CLL is the most common subtype of leukemia in western countries, including the USA (3.9–6.5/100 000 patients) (1), but it is extremely rare in Asia where it accounts for only 0.4%–0.5% of all cases of leukemia (19), and thus represents a disease with significant differences in racial incidence rates (20). The R-FC regimen is still a promising therapeutic option with a favorable prognosis in young and fit patients with CLL. Although oral BTK inhibitors have recently been developed, due to the high cost of new targeted agents and the need to continue administration until progression, R-FC is still an important treatment option ensuring completion to the end of treatment in clinical practice. To our knowledge, this is the first prospective study of the degree of neutropenia/febrile neutropenia occurring after this regimen in an Asian country, and is at least the first such study in Korea.

Our study focused on R-FC chemotherapy for CLL patients. In general, the guidelines for the use of pegfilgrastim re commend that combination chemoimmunotherapy with a febrile neutropenia incidence of 20% or more is classified as high-risk (21). The R-FC regimen is not strictly classified as dose-dense chemotherapy. However, the German CLL Study Group reported a relatively high rate of hematological toxicity in the CLL8 trial, with a rate of hematological toxicity-related FC reduction over 10% (2). Bouvet et al. (15) reported a high rate of hematological toxicity in a community hospital setting where FC dose reductions were common (51.4% of patients). In R-FC regimen-related clinical study conducted at MD Anderson Cancer Center, 927 courses could be investigated out of 224 patients, and among these subjects, grade 3-4 neutropenia was 52% and febrile neutropenia was 25% (6). In another large-scale prospective study, FLAIR trial, grade 3-4 neutropenia and febrile neutropenia were reported in 53.4% and 33.6, respectively (18). Also, the CLL8 trial (2)and ECOG1912 study (22) showed that the incidence of neutropenia associated with R-FC chemoimmunotherapy was as high as 50% or more, to the extent that grade 3-4 neutropenia was reported at 62% and 45%, respectively (**Tables 4**, **5**). In addition, in our historical Asian cohort, hematological toxicity was high during the administration of R-FC chemotherapy (14.7%–85.7%), and the ratio of disrupted chemotherapy was also high (47.3%). Due to the nature of CLL itself, the rates of infectious complications are influenced by the progressive reduction in immunoglobulin levels and are more common in patients with previously treated CLL (13). Taken together, it was suggested that although R-FC itself is not a dose-dense chemotherapeutic regimen, it could be considered a relatively myelotoxic regimen capable of causing therapy-related bone marrow failure and high susceptibility to serious infections, particularly in patients with CLL. Our results indicate significant reductions in rates of neutropenia/febrile neutropenia as well as a decrease in disrupted chemotherapy in patients treated with R-FC with pegfilgrastim prophylaxis. Thus, the use of primary pegfilgrastim prophylaxis can protect against severe neutropenia and neutropenia-related infections in patients with CLL receiving R-FC chemotherapy.

**Table 5:** The incidence of neutropenia in prospective studies related to R-FC.

Study	n	no. of accessible course	neutropenia, Grade 3-4	febrile neutropenia	major infection	reference
MDACC	224	927	52%	25.0%	2.6%	(6)
FLAIR	385	N/A*	53.4%	33.6%	N/A*	(18)
CLL8	404	N/A*	62%	N/A*	3%	(2)
ECOG1912	158	N/A*	45%	15.8%	8.9%	(22)
this research	34	186	9.7%	2.2%	1.1%	–

* N/A, not applicable.

Although a comparison of survival outcomes is not entirely suitable for the purpose of this study, survival analyses were conducted to determine whether there were any changes in survival outcomes as a result of febrile neutropenia reduction and a decreased rate of disrupted chemotherapy with pegfilgrastim prophylaxis. The results indicate no differences in long-term OS, PFS, or CIR in comparison to the historical cohort, but we found a significant decrease in early mortality rate, including death due to a neutropenia-associated infection. Similar to our results, a meta-analysis by Kuderer et al. (23) reported that early mortality was significantly decreased by G-CSF prophylaxis (HR 0.60, 95% CI 0.43–0.83, *P* = 0.002), and Vogel et al. (17) demonstrated superior early mortality control in their G-CSF prophylaxis group (HR 0.359, 95% CI 0.130–0.988, *P* = 0.0.38). These findings indicate the impact of primary pegfilgrastim prophylaxis on infection-related and all-cause early mortality as well as a reduction in disrupted chemotherapy.

Our study showed favorable results of pegfilgrastim prophylaxis in CLL patients receiving R-FC chemotherapy. However, this analysis had several limitations. First, the number of patients enrolled in the study was too small to draw definitive conclusions. In addition, the number of adverse events was small, and the study was not designed and did not have sufficient power to assess OS. Second, the efficacy and survival outcomes of prophylactic pegfilgrastim were examined by comparison of the study cohort with a historical cohort. However, this was not a direct comparison but an indirect incomplete comparison, and therefore care is required in interpreting the results. Third, the follow-up periods were not equivalent among the cohorts (2.6 years in the study cohort and 5.9 years in the historical cohort).

In conclusion, this prospective study suggests that primary pegfilgrastim prophylaxis can reduce the rate of grade 3 or 4 neutropenia/febrile neutropenia in patients with CLL receiving R-FC chemotherapy in contrast to previous studies and our historical cohort in which chemotherapy dose adjustment and infusion schedule delays occurred frequently during R-FC therapy without G-CSF prophylaxis. Moreover, the reduction in the early mortality rate itself suggests that the use of pegfilgrastim prophylaxis is important during R-FC chemotherapy in patients with CLL. Although R-FC is not currently classified as high-risk for febrile neutropenia, considering the characteristics of CLL as an immunosuppressive disease as well as a dose-dense chemoimmunotherapeutic regimen R-FC, prophylactic pegfilgrastim support is an essential adjuvant for R-FC therapy.

## Data availability statement

The raw data supporting the conclusions of this article will be made available by the authors, without undue reservation.

## Ethics statement

The study protocol was approved by the institutional review boards and local ethics committees of all participating centers. The patients/participants provided their written informed consent to participate in this study.

## Author contributions

This manuscript has been seen and approved by all authors, whose individual contributions are as follows: YJ and professor S-GC were contributing to conception and design, acquiring data, analyzing and interpreting data, and drafting the manuscript. YJ and professor S-GC were critically contributing to or revising the manuscript. D-HY, S-JO, J-HP, J-AK, S-YK, C-WC, W-SL, I-HK, Y-CM, GM, and K-SE were enhancing its intellectual content. And S-GC was approving the final content of this manuscript.
